# Genome-wide SNP identification by high-throughput sequencing and selective mapping allows sequence assembly positioning using a framework genetic linkage map

**DOI:** 10.1186/1741-7007-8-155

**Published:** 2010-12-30

**Authors:** Jean-Marc Celton, Alan Christoffels, Daniel J Sargent, Xiangming Xu, D Jasper G Rees

**Affiliations:** 1 Biotechnology Department, University of the Western Cape, Private Bag X17, Bellville 7535, South Africa; 2 South African National Bioinformatics Institute, University of the Western Cape, Private Bag X17, Bellville 7535, South Africa; 3 East Malling Research, New Road, East Malling, Kent ME19 6BJ, UK; 4 Agricultural Research Council, Biotechnology Platform, Private Bag X5, Onderstepoort 0110, South Africa

## Abstract

**Background:**

Determining the position and order of contigs and scaffolds from a genome assembly within an organism's genome remains a technical challenge in a majority of sequencing projects. In order to exploit contemporary technologies for DNA sequencing, we developed a strategy for whole genome single nucleotide polymorphism sequencing allowing the positioning of sequence contigs onto a linkage map using the bin mapping method.

**Results:**

The strategy was tested on a draft genome of the fungal pathogen *Venturia inaequalis*, the causal agent of apple scab, and further validated using sequence contigs derived from the diploid plant genome *Fragaria vesca*. Using our novel method we were able to anchor 70% and 92% of sequences assemblies for *V. inaequalis *and *F. vesca*, respectively, to genetic linkage maps.

**Conclusions:**

We demonstrated the utility of this approach by accurately determining the bin map positions of the majority of the large sequence contigs from each genome sequence and validated our method by mapping single sequence repeat markers derived from sequence contigs on a full mapping population.

## Background

The recent introduction of Next Generation Sequencing platforms such as the Applied Biosystems SOLiD sequencer, the Roche (454) sequencer and the Illumina Genome Analyzer, has seen an exponential increase in genome sequencing efforts for a wide range of organisms. Over the last 2 years, a variety of genomes such as cow [1], papaya [2], cucumber [3] and the filamentous fungus *Grosmannia clavigera *[4], have been sequenced using these platforms. From the short overlapping sequence fragments obtained, it is possible to generate draft genome sequences using various algorithms developed for *de novo *sequence assembly [5-7]. Despite improvements in the software used in the assembly of small DNA sequences, it is very difficult to build a fully assembled genome using short read sequence data alone. The number of contiguous sequences in the final assembly can vary from tens, to several thousands depending on the accuracy of the primary sequence data, the depth of sequence coverage, the length and number of sequence repeats and the genome size of the organism studied.

Various methods have been developed to position sequence scaffolds on physical or genetic maps to assist in the assembly process. Positional information for assemblies can, for instance, be derived from comparison with genomic sequences of related organisms. For relatively small genomes with limited numbers of sequence repeats, gaps between genomic sequences can be bridged by polymerase chain reaction or cloning strategies.

However, these methods remain expensive and time consuming, and are largely impractical for organisms with relatively large genomes. Until now, for organisms where no close relatives had been sequenced, the positioning of sequence contigs relative to one another has required their anchoring to saturated linkage maps which has largely depended upon the availability of abundant mapped genetic markers such as simple sequence repeats (SSR) and single nucleotide polymorphism (SNP) markers.

SNPs are the most common form of genetic variation between individuals, making them very attractive for anchoring genome sequence contigs to linkage maps. Methods for identifying and genotyping these SNPs have developed rapidly in the last few years and, as a result, a variety of SNP genotyping protocols have become available [8]. However, the ability to build high-density SNP assays relies entirely on the current availability of large numbers of SNPs with known genomic coordinates and known allele frequencies. For the majority of species, the development of high-density SNP assays remains a challenge because draft genome sequences, when available, are generated from the DNA of a single inbred individual and, thus, do not readily permit the identification of large numbers of SNPs. Moreover, the cost of complete genome re-sequencing for SNP discovery remains prohibitive for species with large genomes.

A strategy known as 'selective' or 'bin' mapping has been developed, which permits rapid mapping of large numbers of genetic markers to a mapping framework with a low degree of precision, by using only a subset of highly informative progeny individuals [9-11]. Thus, for a given marker, the joint (or combined) genotype of the selected subset of individuals at a locus identifies a unique mapping bin on the genetic map of the organism.

**Figure 1:**
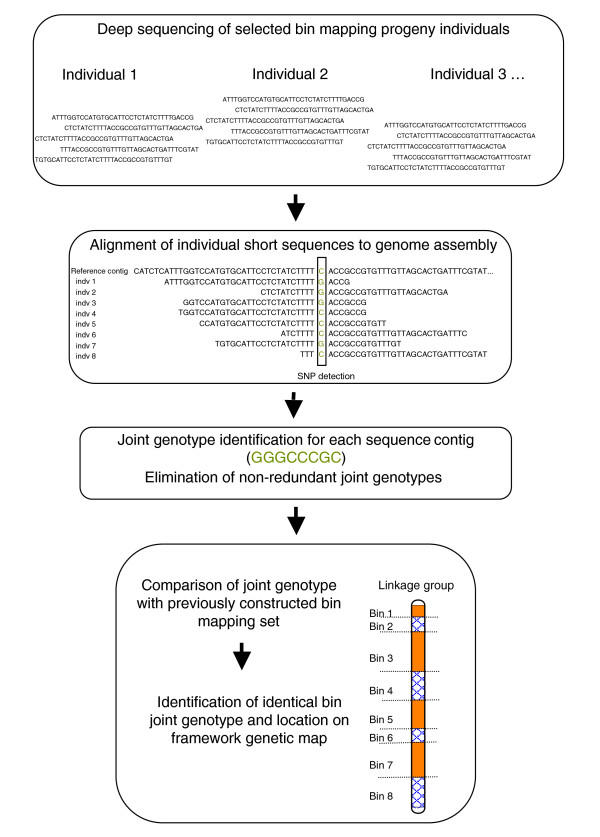
Diagram of the single nucleotide polymorphism using the Sequencing Bin Mapping procedure for anchoring sequence contigs to a framework genetic linkage map.

We describe a method utilizing next generation sequencing to score SNPs and anchor assemblies to a genetic map by exploiting the bin mapping strategy, hereafter referred to as the SNP by Sequencing Bin Mapping strategy (SSBM; Figure 1).

Our approach is based on deep sequencing of genomic libraries from selected progeny individuals. The method was tested on a haploid organism [*Venturia inaequalis *Cooke (Wint.)] and then validated using the diploid plant species *Fragaria vesca *(the woodland strawberry). The genome of *V. inaequalis*, a hemi-biotrophic fungus which is a pathogen of apples (*Malus × domestica *Borkh) [12] has been estimated to be as large as 100 Mbp [13]. However, a draft *de novo *shotgun genome sequence assembled from short single-end and paired-end sequence data, despite having a depth of coverage of approximately 100×, covers just under 40 Mbp of unique sequence (JMC, Hüsselmann L and DJGR, manuscript in preparation). The *F. vesca *genome size, on the other hand, has been estimated to be in the region of 206 Mb [14]. Sequencing of the *F. vesca *genome has recently been carried out using a range of sequencing platforms [15]. *De novo *assembly yielded a total of over 3200 scaffolds, of which 272 cover 209.8 Mb.

Using a bin mapping set derived from a genetic linkage map, whole genome re-sequencing was performed on individuals issued from a *V. inaequalis *population. The validation of our strategy was then performed by re-sequencing individuals derived from a cross between two closely related diploid strawberry species *F. vesca *and *F. nubicola *[16]. The relative large size of the *Fragaria *genome meant that re-sequencing was performed on reduced-complexity libraries in order to maximize the genome coverage at particular locations. The overall objective was to maximize the number of SNPs detected by whole-genome, or partial re-sequencing of selected progeny individuals, and to use the SNPs identified to anchor the draft genome assembly to an existing genetic map using a bin mapping strategy.

## Results and discussion

### Test of the SSBM strategy on *V. inaequalis*

#### Genome sequencing and assembly

**Figure 2:**
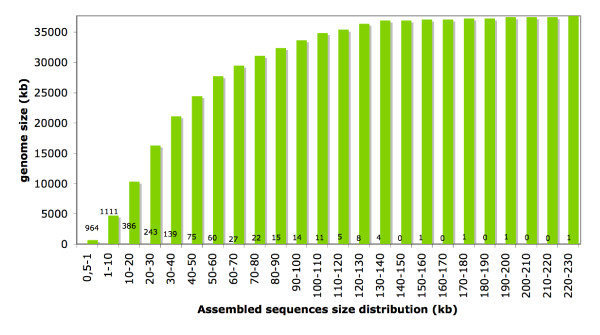
**Cumulative distribution of assembled sequences from the *Venturia inaequalis *genome**. Sequences were assembled using Velvet [7], from a mixture of single-end and paired-end short sequences generated by the Illumina Genome Analyzer II. Numbers located along the x-axis of the graph represent the number of assembled sequences per category.

Sequencing of the *V. inaequalis *genome has recently been performed (JMC, Hüsselmann L and DJGR, manuscript in preparation) using Illumina sequencing technology. *De novo *assembly of a mixture of single- and paired-end sequences was performed using Velvet [7] and yielded a total of 3088 sequence contigs larger than 500 bp. These sequence contigs totalled 37,685,262 bp with a median size of 2817 bp, an average size of 12,204 bp and a largest sequence assembly size of 220,681 bp. More than 85% of the genome sequence contigs were longer than 10 kb (Figure 2).

#### Construction of the bin mapping set

Using the selective mapping strategy, eight haploid progeny isolates were selected from a population derived from a cross between isolates C1054 (China) and 01/213 (UK). The framework linkage map developed for this progeny [17], which covers a total of 804 cM, was divided into 54 bins using eight individuals following the method described previously [11]. The average bin length was 15.5 cM and the longest bin length identified was 31.7 cM. As there were a limited number of individuals selected and because of the haploid nature of the organism under investigation, 13 non-contiguous pairs of bins mapping at different locations in the genome were identified as having identical joint genotypes.

#### Library sequencing

The small genome size of *V. inaequalis*, coupled with recent improvements in read length and throughput on the Illumina Genome Analyzer platform, have made it possible to use whole genome re-sequencing for SNP discovery and validation. Thus, the genomes of all eight individuals in the bin mapping set were sequenced to a depth in the range of 2× and 6.2×. The total amount of sequence data obtained for each of the eight libraries varied from 80 (library 5) to 251 Mb (library 6; Table 1). Of the 50 bases generated for each sequence, only the first 35 bases were used for sequence alignment and SNP detection. As shown by the Illumina Solexa output files, the raw quality of the bases sequenced following base number 35 started decreasing significantly. Therefore, to avoid the identification of false SNPs and the identification of false joint genotypes, the last 15 bases of each sequence were not taken into consideration.

**Table 1:** Library sequencing from total DNA of eight selected *Venturia inaequalis *individuals from the mapping progeny C1054 × 01/213.

Individual	Number of sequences	Final volume of data used for analysis (Mb)
1	6,596,811	231
2	6,796,360	238
3	6,843,864	239
4	6,792,617	238
5	2,283,128	80
6	7,181,955	251
7	6,973,366	244
8	4,050,540	141

The 'number of sequences' represents the total number of short sequence fragments sequenced for each individual.

#### Sequence alignment and joint genotype detection

Alignment of the 35 bp sequences to the reference *V. inaequalis *genome was performed with MAQ [18] using the stringent conditions described in the methods section. Under these conditions, putative SNPs were identified in 2623 of the 3088 unique sequences (85%). High quality SNPs were identified, on average, every 585 bp.

A total of 1232 contigs were identified with at least three joint genotypes each and represented 36,946,609 bp, or 98%, of the assembled genome sequence used in the analysis. The number of identical joint genotypes identified per sequence assembly varied from three to 87, with an average of 10 and a median of seven.

As the *V. inaequalis *genome sequence used as the reference was different from the two parental strains used in the construction of the bin mapping progeny, the validation of the joint genotype for each sequence assembly was confirmed visually in to avoid the detection of false positive SNPs and priority was given to sequence contigs with a length in excess of 20 kb.

#### Contig anchoring and validation

Of the 627 sequence contigs which were larger than 20 kb, 514 (82%) were identified as having an unambiguous joint genotype and, thus, could potentially be anchored onto the genetic map. In addition to these, the joint genotype of an extra 440 sequence contigs with a sequence length shorter than 20 kb was also scored. This brought the total number of sequence contigs that could be scored for SNPs in the bin mapping progeny to 954, covering 28,045,768 bp (74%) of the draft genome assembly.

**Figure 3:**
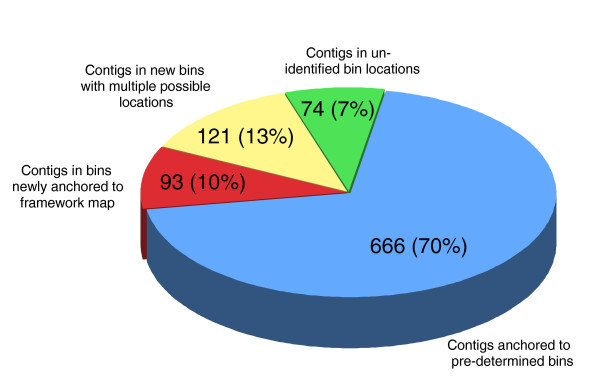
**Anchoring of the 954 sequence contigs for which a joint genotype was identified at least three times within each contig**. The number and percentages are indicated for each bin category (pre-determined bins, newly anchored bins, new multiple location bins, un-identified bin location).

The joint genotype of each of the 954 sequence contigs was then compared to the joint genotype of the pre-determined bins. From this analysis, 666 sequence contigs (70%) were anchored to pre-determined bins, with the remaining 288 (30%) falling into bins that had not previously been identified (Figure 3) because of the restricted marker density on the original linkage map, which was estimated to cover around 70% of the *V. inaequalis *genome. These new bins were analysed and their position on the linkage map was determined by comparing their joint genotypes with the joint genotypes of neighbouring bins, in between which more than one recombination event was observed. and with the joint genotypes of bins located at the extremities of the linkage groups. This comparison allowed the positioning of 17 new bins (93 sequence contigs) on the linkage map. We also established that eight additional new bins (121 sequence contigs) could be positioned at more than one location in the bin map. Details of the analysis are presented in Table 2. Finally, no potential location could be identified for 19 new bins (74 sequence contigs) which, presumably, lie outside the framework of the current linkage map.

**Table 2:** Analysis of the *Venturia inaequalis *genome sequence contigs anchored to the genetic map.

Sequence contigs size (kb)	Total number of sequence contigs	Sequence contigs scored and percentage (%)	Sequence contigs anchored and percentage (%)
< 20	2461	440 **(17.9)**	353 **(14.3)**
20-30	243	175 **(72.0)**	156 **(64.2)**
30-40	139	111 **(79.9)**	75 **(54.0)**
40-50	75	68 **(90.7)**	54 **(72.0)**
50-60	60	54 **(90.0)**	41 **(68.3)**
60-70	27	26 **(96.3)**	20 **(74.1)**
70-80	22	22 **(100.0)**	17 **(77.3)**
80-90	15	14 **(93.3)**	9 **(60.0)**
90-100	14	14 **(100.0)**	9 **(64.3)**
100-110	11	11 **(100.0)**	10 **(90.9)**
110-120	5	4 **(80.0)**	3 **(60.0)**
120-130	8	7 **(87.5)**	6 **(75.0)**
130-140	4	4 **(100.0)**	3 **(75.0)**
> 140	4	4 **(100.0)**	3 **(75.0)**

The sequence contigs anchored comprise the contigs for which the joint genotype matches the joint genotype of the pre-determined bins and the contigs for which the bins could be placed into the map. The number of sequence contigs scored and anchored is followed by their respective percentage relative to the total number of sequence contigs indicated in the second column.

In order to validate our strategy, the map location of 48 SSR markers identified from the *V. inaequalis *genome sequence (Additional File 1) was compared to the bin location of the sequence contigs from which they had been developed. Of these 48 sequence contigs, 18 were not bin mapped by SNP-seq because the number of joint genotype identified in the sequence did not reach the threshold of three required for validation. Of the remaining 30 sequence contigs, 26 were located in the bin in which their respective SSR had been mapped and four were misplaced. Primer sequences for the 48 SSR sequences are given in Additional File 2. A detailed analysis of the output of the MAQ program revealed that these sequence contigs had been misplaced, mainly because of the low coverage observed for library 5 (Table 1).

### Validation of the SSBM strategy on *F. vesca*

#### Bin mapping set construction

Six F_2 _individuals were selected from a mapping population derived from a cross between *F. vesca *and *F. nubicola*. The linkage map constructed from this cross covered a total distance of 545 cM and was divided into 55 bins.

#### Library sequencing

DNA fragments of 74 bp were generated by the Illumina Genome Analyzer II, of which 63 bp were used for sequence alignment. Considering that the Alu I fragments isolated represented approximately 10% of the genome, each library thus presented a total coverage of 4.8× to 48× at these particular genomic locations. The final size of data used for the analysis varied from 159 to 1002 Mb (Table 3).

**Table 3:** Library sequencing from reduced complexity DNA libraries derived from six selected individuals from the mapping population *Fragaria vesca *x *F. nubicola*.

Individual	Number of sequences (bp)	Final volume of data used for analysis (Mb)
Parent *F. nubicola*	11,452,620	721
1	15,388,017	969
2	9,125,899	575
3	2,521,718	159
4	14,805,664	933
5	15,920,025	1002
6	1,584,695	100

#### Sequence alignment, scaffolds anchoring and validation

Alignment of the 63 bp sequences to the *F. vesca *genome was performed using the same stringent conditions used previously. Using sequences derived from the male grand-parental library (*F. nubicola*), SNPs were identified in the majority of the 3200 scaffolds and one SNP was detected in approximately 25% of the Alu I fragments. Using our approach, 185 of the 211 scaffolds of over 100 kb in length of the *F. vesca *genome sequence were anchored to the genetic map (Table 4). In addition, 11 scaffolds ranging in size from 4470 to 83,161 bp were also anchored. In total, 92.8% of the *Fragaria *genome was anchored to the genetic map using the SSBM strategy.

**Table 4:** Detailed presentation of *Fragaria *contigs over 100 kb scored and anchored to the genetic map, per size range.

Contig size (kb)	Number of contig assemblies	Contigs anchored (%)	Total size anchored (kb)
100-500	88	70 (79.5)	20172
500-1000	58	54 (93.1)	38113
1000-2000	35	32 (91.4)	44189
2000-3000	20	20 (100)	49860
3000-4000	6	5 (83.3)	17283
4000-5000	1	1 (100)	4105
5000-6000	2	2 (100)	10912
> 6000	1	1 (100)	6096

Total	211	185 (87.7)	190730 (90.9)

The number of contigs anchored is followed by their respective percentage (in bold) relative to the total number of contig indicated in the first column. The last column indicates the total size in kb anchored to the map.

Validation of the contig location was performed by mapping SSR and SNP markers developed from *Fragaria *sequences. Of the 113 markers developed, 95 (84.1%) were mapped to genomic regions corresponding to the bin location where their respective scaffolds had been anchored. The majority of the remaining markers were mapped to genomic regions corresponding to adjacent bin locations.

In a few cases, SNP identification at regular intervals along *Fragaria *sequence assemblies allowed us to identify clear changes in joint genotypes within the same assembly. For six of these assemblies, this change in joint genotype did not correspond to a passage from one bin to the next but to a completely different bin located in another part of the genome. Detailed analysis of the assemblies using the SNP data allowed us to pinpoint the location in which the miss-assembly had occurred, thus improving the final quality of the assembled sequence.

## Conclusions

Our strategy has shown that it is now possible to anchor genome sequence contigs to a reference linkage map without having to first develop and locate large numbers of sequence characterized genetic markers or undertake the sequencing of bacterial artificial chromosome (BAC) ends. This method is ideally suited to organisms for which genetic resources are poorly developed. Using a foundation genetic map, we have developed the possibility of anchoring and ordering genome sequence contigs rapidly, easily and cost-effectively, without the need of prior extensive genetic knowledge of the organism studied.

The anchoring of sequence contigs to bins provides, in most instances, an approximate estimate of the location of a sequence in a particular linkage group but does not allow the relative positioning of sequence contigs within each bin. Thus, the accuracy of the location of each sequence assembly within the map depends entirely on the initial selection of the individuals to be sequenced and on the size of the sequence contigs to be anchored. Sequencing more individuals would eventually eliminate bins with similar joint genotypes and permit the identification of additional bins, therefore leading to an increased precision of the bin mapping. However, the location of large numbers of sequence contigs within an existing genetic map can serve as a robust framework for the anchoring of the genome sequence of an organism without the requirement of a high density physical mapping or well-saturated genetic mapping platform. Once scaffolds have been located to mapping bins, the precise positioning of scaffolds and their orientation relative to the linkage map can be performed in a targeted fashion. Where more precise positioning and orientation is required for a given genome region, individual SNPs can be scored in the entire mapping population from which the bin set was derived and scaffolds can be located relative to the genetic markers used to generate the foundation linkage map.

For larger haploid or diploid genomes, the SNP mapping by sequencing strategy can also be applied, as it is possible to sequence a large number of SNPs across all selected progenies by targeting specific regions of the genome. As demonstrated by our study performed on the diploid genome of *F. vesca*, this can be achieved by constructing libraries with a reduced complexity using restriction enzymes cutting at frequent intervals throughout the genome.

Furthermore, the SSBM strategy can also contribute to improve the quality of the genome assembly by identifying misassembled fragments through the identification of SNPs at regular intervals. Thus, our strategy provides a way to validate the assembly of large sequence fragments.

As a result of the very high throughput generated by the Illumina Genome Analyzer II, libraries could in the future be indexed and run together within a single lane of a flowcell, thus reducing the initial sequencing costs. Conversely, provided that the organism studied has a relatively small genome size, whole sequencing of a subset of individuals derived from a mapping population, instead of a single inbred individual, could provide sufficient data for the *de novo *assembly of the organism's genome and for the positioning of sequence contigs derived from the sequence assembly into a genetic map.

Given the flexibility and scalability of this approach, together with its demonstrated power to detect large numbers of high quality SNPs, we expect this method to significantly reduce the time and costs associated with *de novo *sequencing and alignment of sequence contigs to a linkage map. In addition, it should be possible to undertake this process without the use of an existing linkage map. For relatively small genomes, the sequencing of a larger set of individuals could provide enough data to perform *de novo *sequence assembly, SNP identification and relative sequence assembly positioning. The use of long mate pair sequencing will result in the generation of much larger *de novo *assemblies which will, in turn, allow the use of reduced complexity sequencing to provide the SNP-seq bin set data needed for the application of the SSBM strategy to larger genomes. This opens the way for the generation of low cost draft genome sequencing for a wide range of minor crops and pathogens for which funding is usually limited.

## Methods

### Genome sequencing

#### *V. inaequalis*

DNA was isolated from eight individuals derived from a bin mapping set using a protocol developed by [19] with modifications from [20]. Purified DNA was then further prepared according to the manufacturer's protocol (Illumina GAII analyzer). Each library was run on a separate GAII lane in order to obtain a maximum coverage of the genome for each individual.

#### *Fragaria*

Six F_2 _individuals derived from a cross between *F. vesca *and *F. nubicola *were selected to form the bin mapping set. The larger genome size of this species meant that reduced complexity libraries were constructed using the restriction enzyme Alu I. As for *V. inaequalis*, purified DNA was further prepared according to the manufacturer's protocol. Libraries were run on separate GAII lanes.

### Sequence alignment to reference genome and joint genotype identification

Alignment of the sequences to their respective reference genome was performed with MAQ [18] using stringent conditions, based on high base quality values (maq assemble: -*m *= 2; -*Q *= 70 -*q *= 20), and using only sequences aligning to the reference genome with less than two mismatches.

Putative SNPs obtained from MAQ were examined over all the selected individuals in order to generate joint genotypes as follows: SNPs identified in the individuals were scored relative to the SNPs at identical positions in the reference strain in order to generate a joint genotype at different locations on the assembled contigs. Contigs were retained for further screening, provided they contained at least three occurrences of the same joint genotype.

## Abbreviations

SNP: single nucleotide polymorphism; SSBM: SNP sequencing Bin mapping; SSR: simple sequence repeat.

## Authors' contributions

JMC designed the experiment, sequenced and analysed the libraries and prepared the manuscript. AC designed the PERL script used to extract SNP data. XX and DJS provided the bin mapping population and mapped the SSR markers used in the validation of the method. DJGR was project leader and contributed to the experiment design.

## Supplementary Material

Additional file 1 **Supplementary Figure 1**. The map positions of simple sequence repeat (SSR) markers identified from *Ventura inequalis *genome sequence scaffolds on the updated map of Xu *et al. *[18]. The map was constructed using the methods and data of Xu *et al. *[18] with the addition of data for 48 SSRs scored in the full progeny. In total, 45 SSRs located to positions on the seven *V. inequalis *linkage groups, whilst the three remaining markers were unlinked. Click here for file

Additional file 2 **Supplementary Table 1**. Primer sequences and expected product sizes for 48 single sequence repeats (SSRs) used to locate *Venturia inaequalis *sequence scaffolds to the linkage map of Xu *et al. *[18]. Click here for file
