# Supplementary figures and images for: Genome-wide SNP identification by high-throughput sequencing and selective mapping allows sequence assembly positioning using a framework genetic linkage map
Source: BMC Biol. 2010 Dec 30;8:155. doi: 10.1186/1741-7007-8-155 (PMC3024234; doi:10.1186/1741-7007-8-155)

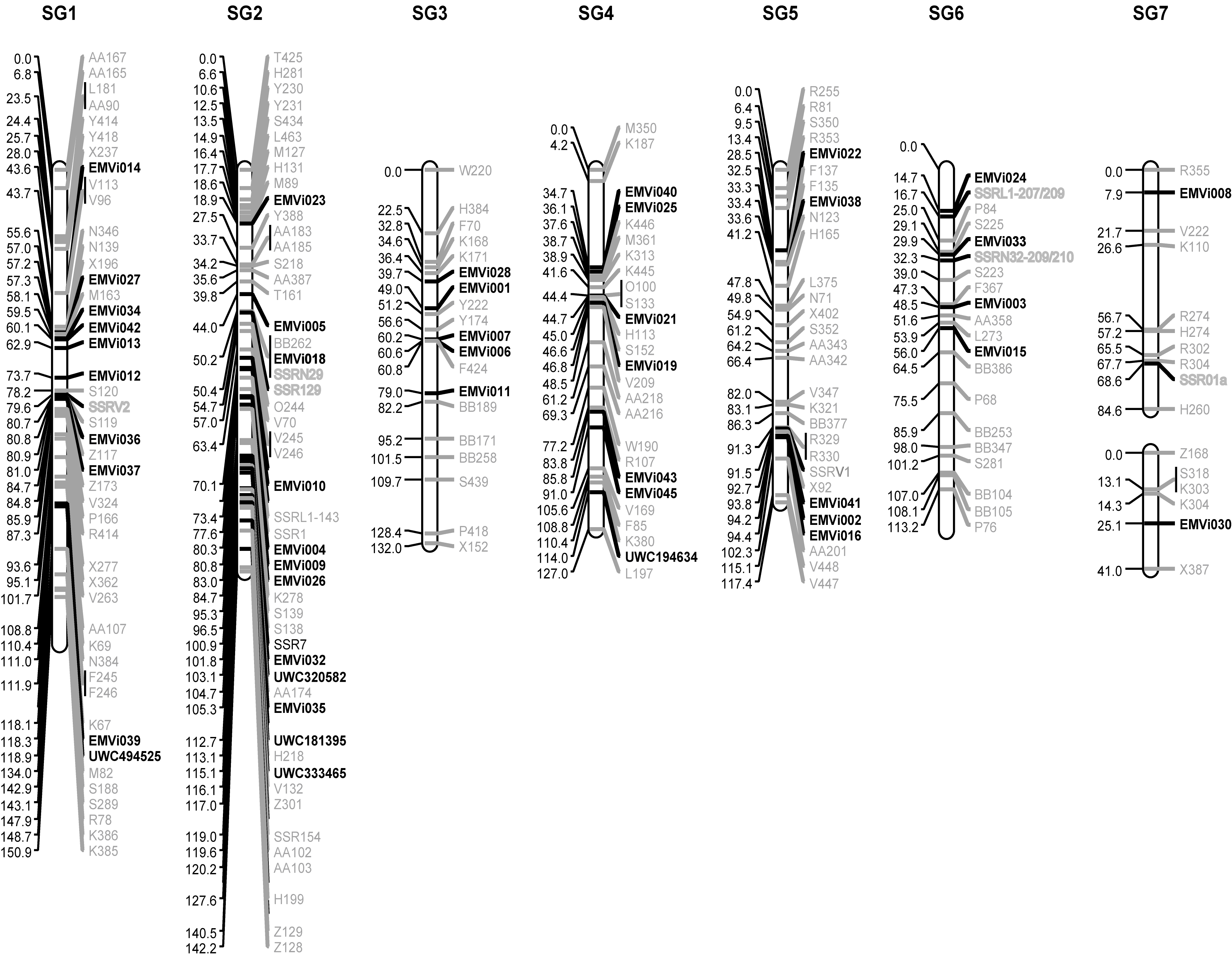

Supplement: Additional file 1 — Supplementary Figure 1. The map positions of simple sequence repeat (SSR) markers identified from Ventura inequalis genome sequence scaffolds on the updated map of Xu et al. [18]. The map was constructed using the methods and data of Xu et al. [18] with the addition of data for 48 SSRs scored in the full progeny. In total, 45 SSRs located to positions on the seven V. inequalis linkage groups, whilst the three remaining markers were unlinked. [file 1741-7007-8-155-S1.png]
