# Supplementary material for: Genome-wide SNP identification by high-throughput sequencing and selective mapping allows sequence assembly positioning using a framework genetic linkage map
Source: BMC Biol. 2010 Dec 30;8:155. doi: 10.1186/1741-7007-8-155 (PMC3024234; doi:10.1186/1741-7007-8-155)
Supplement: Additional file 2 — Supplementary Table 1. Primer sequences and expected product sizes for 48 single sequence repeats (SSRs) used to locate Venturia inaequalis sequence scaffolds to the linkage map of Xu et al. [18]. [file 1741-7007-8-155-S2.doc]

**Supplementary Table 1**

| Locus name | Forward Sequence | Reverse Sequence | Expected Size |
| --- | --- | --- | --- |
| EMVi001 | AATTAGTCCAACAAGGCTTTCC | ACGTGGGTGTAGGTACTCAAGG | 234 |
| EMVi002 | TTTGTTGTAGCTAGCTGTGTTCG | GCCAGACTCCAGAATAAAAAGG | 128 |
| EMVi003 | CCCTTGTAAAGGACGAAAAGG | ATGATTCTGCTCCTCTCTCTCG | 145 |
| EMVi004 | CGCAGACTCGTCCATTATTACC | ATCATCATCGTCTTTCATCACG | 388 |
| EMVi005 | TGGTGTTGATGAAATTGAGAGG | TAACACCAGTACTTCCGCTTCC | 161 |
| EMVi006 | AGGTGAGATTGTGTGATTCTGC | AGACTTGGGTATTGGATGATGG | 172 |
| EMVi007 | CAGCCATGATTTTCTACAGTCG | AAAACGAGTTCTAGGCTGTTGG | 369 |
| EMVi008 | GAATCATGAAGCGAAAAAGAGG | ATAGTGATCGCCAAGAAAAAGG | 314 |
| EMVi009 | CTGTACAACGTACGACCTGTGC | GTCCTGTCCTTGTTCGTAGTCG | 362 |
| EMVi010 | TAATGCGGTTATGTTGAAGC | CTCGTCTCCTCTCCTCTTCTCC | 169 |
| EMVi011 | GAAAGACGTCGTGATTTTCTCG | ATGCAAAGCATATGGATCTGG | 221 |
| EMVi012 | AAGATGTGTATATGCGGGATGG | TACATACATGGGCAGAAGAAGG | 378 |
| EMVi013 | TTAATACCTAGGCCGTTATACGC | AGAAGTAGACCCCCGAAGTAGG | 349 |
| EMVi014 | CATTTGCTGGAGTCGGATGG | AGGAACGCGTTGAATTATTGG | 247 |
| EMVi015 | ACCAGGTATGTCCATCAATTCC | ACTCTCCTTCCTGGCTTACAGG | 130 |
| EMVi016 | GGAAGGGAAGGAGAAAGAGG | CATGAGCAGAGAACGAGTTTCC | 147 |
| EMVi018 | GGACCTCTCGTTTCATGAGG | CTCTCTCTCTTCTTCCATTCTGG | 121 |
| EMVi019 | GATCTAGGGAATCAAGCACTGG | CGGGTCGTATAGGTAGAGTTGG | 152 |
| EMVi021 | CTTACATCCGAGAGGTCACTCC | AGCGCCAAATAAGAAAGAAACC | 220 |
| EMVi022 | GTGGTTATGTTGTGGGAGTGG | GTACTCTCTCGGCCTAAACTCG | 230 |
| EMVi023 | GGGAGGGTTGGTTAGTGAAGC | AGACAGAGGAGGAAAATCATGG | 189 |
| EMVi024 | GTGAGGATAGGGTTGGTGAAGG | GCGTCTTCGAAAGGATATATGG | 179 |
| EMVi025 | CCACCCATACCTTTAGAACTGC | GGGAAGAATGTAAAAAGCATCC | 229 |
| EMVi026 | TCGATGGATATGACATCTACGC | GGTGATTTCTTGGTTTTCACG | 156 |
| EMVi027 | AATTCGGGTCGGTATAAGTAAGC | CATTTCGTCCTATGGTGGTACG | 192 |
| EMVi028 | TAATTGAACAGGGTCTCCAACG | CACAACATGATGAAAACGAAGG | 204 |
| EMVi030 | TCACGACACTGACATTCAAAGC | GGTTGGTTGTTGGTATACTTTCG | 189 |
| EMVi032 | GATTAATCTGTAGCGCGTTTCC | TTTTCTCGTAAAGTCGTCATGC | 322 |
| EMVi033 | AATGTCTGCAAACCCTTATTGC | CTTTTCTGGTTTGGTTTTGTCC | 316 |
| EMVi034 | GTTGTGGCTTCATCTCCATACC | TTTTAGATTGGTGCTGTTGTGG | 251 |
| EMVi035 | CCATACTCGTCTGAACACTTGG | GGACTTCTATCCAGTCCAAAGC | 282 |
| EMVi036 | TGCTCTACATCTCATCCCATCC | TCCTTCCTTCAACAAGATAGGC | 264 |
| EMVi037 | GGAAAGGAACACGATTATCAGG | GAATGGGGATATTGAGAAGAGC | 313 |
| EMVi038 | GCAAGGAATAAGGATAATAGGC | GGGCTAGCCTAGAAATAAAAGACC | 200 |
| EMVi039 | GTTCCAGCACAACGTCTACACC | GTTCAAACCTCTCTGAAAGTCG | 140 |
| EMVi040 | GACATCTCTCCCATCTCAGAGC | GTCGAGTCTGAAATGCATGG | 149 |
| EMVi041 | CGTAACCATCACCTCTATCACG | TGATATGGAGAACCATGAGTGG | 337 |
| EMVi042 | AAACTCAACGTCAAGGAGAGG | ACCTCGCCCTCCAACTTTCC | 120 |
| EMVi043 | CTTTTCCAAAGTCGAGAGTTCC | GTTTCTTGGTAGGCAGATTTGG | 418 |
| EMVi045 | GGCAAGATGTGAGTATGGAAGC | ATGGCAGCCTATAACAAAGAGC | 265 |
| EMVi076 | ATCGCCAAAGGACAAAGACC | GCAACTCTCTTTGCTGTGAGC | 102 |
| EMVi077 | ACCCGGTCGTAGATTAGAAGC | CCTCCACACCAGTATTCTTTGG | 209 |
| EMVi078 | GAAGGGTGTTAAGTGAGGATGG | CCTCCGAAGGCTTCATCTCG | 134 |
| UWC181395 | CGTGGGAAATGAGTCCAAGT | TGTGGACTACCACCAACCAA | 140 |
| UWC194634 | CACCACAACGACCGAAATAA | TTGGTCACGTTGATTTTTGC | 316 |
| UWC320582 | AGGAAGTCGCCGAAGTTGT | TGAGGAGGCTAGCTACGTTGT | 220 |
| UWC333465 | CGACTTCAAGGGGATTGTTG | ACTCCAGAGACGGCGAAGTA | 179 |
| UWC494525 | GTATGTGCTGATGCCTGGTG | TACAACCAGAGATGCGATGG | 121 |
